# Development and Preclinical Evaluation of a Lyophilized Vaccine Against Equine Herpesvirus Type 4 (EHV-4)

**DOI:** 10.3390/vaccines13060604

**Published:** 2025-05-31

**Authors:** Lespek Kutumbetov, Balzhan Myrzakhmetova, Aiganym Tussipova, Gulzhan Zhapparova, Talshyn Tlenchiyeva, Karina Bissenbayeva, Sergazy Nurabayev, Aslan Kerimbayev

**Affiliations:** Research Institute for Biological Safety Problems, Gvardeiskiy 080409, Kazakhstan; lespek.k@gmail.com (L.K.); aiganym.t24@gmail.com (A.T.); gulzhan1003@mail.ru (G.Z.); t.m.tlenchieva@mail.ru (T.T.); bisenbayeva.karina@bk.ru (K.B.); sergazy-75@mail.ru (S.N.); aslan_kerim@mail.ru (A.K.)

**Keywords:** equine herpesvirus type 4, lyophilized vaccine, EHV-4 vaccine, thermostability, immunogenicity, vaccine development

## Abstract

Background/Objectives: Equine rhinopneumonia, caused by equine herpesvirus types 1 and 4 (EHV-1 and EHV-4), continues to be a significant health and economic concern in the global equine industry, particularly in Kazakhstan. While vaccines targeting EHV-1 are available, there is currently no licensed monovalent vaccine for EHV-4, and existing formulations offer limited protection against this serotype. This study aimed to develop and evaluate a freeze-dried, live-attenuated EHV-4 vaccine with improved safety, stability, and immunogenicity. Methods: A field isolate of EHV-4 was attenuated through serial passaging in primary lamb testicle (LT-KK49) cell cultures. Viral biomass was concentrated and formulated with various stabilizers before freeze-drying. The most effective stabilizer composition—sucrose, gelatin, and lactalbumin hydrolysate—was selected based on viral titer retention. Safety and immunogenicity were assessed in mice, guinea pigs, rabbits, donkeys, and horses. A guinea pig reproductive challenge model was used to evaluate protective efficacy. Results: The optimized lyophilized vaccine retained infectivity (>6.0 log_10_ TCID_50/cm_^3^) for at least six months at 4 °C. No adverse clinical signs were observed in any test species. Immunization induced robust neutralizing antibody responses in both small animals and equines. In the guinea pig model, vaccinated females demonstrated 100% pregnancy retention and fetal viability following challenge with a virulent EHV-4 strain. Conclusions: This freeze-dried, live-attenuated EHV-4 vaccine candidate is safe, immunogenic, and thermostable. It offers a promising platform for the targeted prevention of EHV-4 infection, particularly in young horses and in regions with limited cold-chain infrastructure.

## 1. Introduction

Equine rhinopneumonia is a highly transmissible viral affliction that impacts horses of all ages, resulting in respiratory sickness, abortions, and, in certain instances, neurological abnormalities [1,2]. The condition is attributable to two closely related pathogens: equine herpesvirus type 1 (EHV-1) [3,4,5,6] and equine herpesvirus type 4 (EHV-4) [7,8,9], both of which are part of the Alphaherpesvirinae subfamily. EHV-4 is the predominant cause of acute respiratory sickness, especially in foals and young horses, but EHV-1 is more commonly linked to abortion storms and equine herpesvirus myeloencephalopathy (EHM) [3,10,11,12,13].

Global outbreaks of rhinopneumonia have been documented [14,15,16,17,18], leading to considerable economic losses attributed to veterinary expenses, diminished performance, quarantine measures, and reproductive failures. The disease is especially concerning in the Republic of Kazakhstan, where equine breeding is a culturally and commercially significant agricultural sector [19,20].

Notwithstanding the accessibility of vaccinations, horse herpesvirus continues to be challenging to manage. Currently, the majority of vaccinations are formulated utilizing either inactivated or modified live EHV-1 strains, frequently in conjunction with EHV-4 in a bivalent vaccine [21,22,23]. The protective effectiveness of these formulations against EHV-4 is inadequate. They may mitigate the intensity of clinical manifestations, but may not avert infection, viral shedding, or reactivation, especially with EHV-4 [24]. Moreover, the immunity provided is transient, necessitating regular revaccination, and as yet there are no vaccines that only target EHV-4.

Making a safe, effective, and heat-stable vaccine for EHV-4 is an important step in improving the prevention of rhinopneumonia, particularly in breeding farms and young horses where EHV-4 is common. This paper explains how a freeze-dried EHV-4 vaccine was made from a changed harmless strain grown in lamb testicle cell culture, and it discusses its stability and testing before clinical use. The vaccine underwent evaluation for safety, immunogenicity, and thermal stability in both small laboratory animals and equines. This vaccine candidate targets a specific disease and presents an optimized formulation for prolonged storage, addressing the significant constraints of existing choices and potentially providing practical benefits for field use in Kazakhstan.

## 2. Materials and Methods

### 2.1. Ethical Approval

The study was executed in compliance with ethical norms and standards, including the Guidelines from the Council of Europe Convention for the Protection of Vertebrate Animals Utilized for Experimental and Other Scientific Purposes; the Directive from the Council of the EAC; and Suggestions from the FELASA Working Group Report (1994–1996) [25,26].

### 2.2. Virus Attenuation and Cell Line Selection

To find the best surface for EHV-4 to grow, the virus was repeatedly transferred to different cell types, including LT (lamb testicle cells), SPEV (swine embryo kidney cells), MDBK (Madin–Darby bovine kidney cells), and DCE (developing chicken embryo cells). All cell cultures were provided by the Cell Biotechnology Laboratory of the Research Institute for Biological Safety Problems. The effectiveness of viral replication in each substrate was evaluated based on the degree of cytopathic effect (CPE), replication rate, and final viral titers, as determined by TCID_50_ assays.

CPE in susceptible cell lines was characterized by cell rounding and swelling, the formation of intercellular gaps, and detachment from the culture surface. The LT-KK49 cell line showed the most viral growth and steady cell damage among the tested options, so it was chosen to make the vaccine material and for further weakening processes.

### 2.3. Molecular Identification of EHV-4 by Real-Time PCR

The virus’s identification was verified by real-time PCR (qPCR). Viral DNA was isolated from the culture supernatant utilizing the innuPREP DNA Mini Kit 2.0 (IST Innuscreen GmbH, Berlin, Germany). The test was performed using the genesig^®^ Std Real-Time PCR Detection Kit for Equid Herpesvirus 4 (PrimerDesign, Chandler’s Ford, Hampshire, UK), following the instructions from the manufacturer. Reactions were carried out on a QuantStudio 5 Real-Time PCR System (Thermo Fisher Scientific, Waltham, MA, USA), using special primers and probes that detect the EHV-4 genome through fluorescence. 

Cycle threshold (Ct) readings within the recognized diagnostic range validated the existence of EHV-4. Each run had positive and negative controls to guarantee specificity and eliminate contamination.

### 2.4. Vaccine Production, Stabilization, and Lyophilization

#### 2.4.1. Viral Biomass Preparation

The virus was grown in LT-KK49 cells that were kept in Eagle’s minimal essential medium (Merck, Darmstadt, Germany) with 10% fetal bovine serum (FBS). Viral inoculation was conducted at a multiplicity of infection (MOI) optimized for peak production.

The fluid containing the virus was collected when it caused noticeable damage to at least 80% of the cell layer, usually 72–96 h after infection. The harvested supernatant was cleared via low-speed centrifugation to exclude cellular debris, and was thereafter kept at −80 °C before formulation.

#### 2.4.2. Vaccine Formulation and Lyophilization

The purified virus solution was transformed into a liquid vaccination using a protective stabilizing agent. This medium comprised the following components: sucrose (3–5% *w*/*v*), peptone (3%), gelatin (1.5–2%), lactalbumin hydrolysate (2–2.5%), and lactose (3–4%) (Sigma Aldrich, Saint Louis, MO, USA).

We tested different mixes of these stabilizers to find the best formula for keeping the virus active during the freeze-drying process. Vaccine aliquots (1.0 cm^3^) were dispensed into sterile glass vials under aseptic conditions. Vials were partially sealed with rubber stoppers and placed in a programmable freeze-dryer. The lyophilization cycle comprised the following: initial freezing at −40 °C for 4–6 h; primary drying under vacuum at −50 °C for 24–36 h; and secondary drying at slightly increased temperatures (up to +20 °C) for moisture elimination.

Subsequent to drying, vials were completely stoppered and sealed under vacuum to preserve sterility and stability. The lyophilized vaccine was maintained at 4 °C until required for use.

### 2.5. Biosafety and Immunogenicity Testing

#### 2.5.1. Sterility Testing

Sterility was evaluated in compliance with GOST 28085-89 [27]. Reconstituted vaccination samples were added to the following growth media: meat peptone broth (MPB), meat peptone agar (MPA), meat peptone glucose broth (MPGB), Sabouraud agar (for fungi), and thioglycollate broth (for anaerobes and quick testing).

Samples were cultured under suitable conditions (28–37 °C) for a duration of up to 15 days. A sample with established contamination functioned as a positive control. The lack of microbial development signified sterility.

#### 2.5.2. Safety (Harmlessness) Testing

The vaccine’s safety was assessed in the following subjects: white mice (15 subjects, 18–24 g), guinea pigs (6 subjects, 350–400 g), rabbits (6 subjects, 2.0–2.3 kg), donkeys (5 subjects, 120–150 kg), and horses (4 mares, 300–400 kg).

Animals were administered the immunizing dosage 2–3 times via intramuscular injection and monitored daily for 14 days. Body temperature, local reactions at the injection site, behavioral and appetite changes, mortality, and morbidity were monitored.

#### 2.5.3. Immunogenicity Testing

The vaccine’s ability to trigger an immune response was tested using two methods: the virus neutralization test (VNT) in LT-KK49 cell cultures, and the Hemagglutination Inhibition (HI) test in 96-well plates with horse and rooster red blood cells (specific to guinea pigs).

Serum samples were obtained from guinea pigs prior to and 14 days after the challenge. Samples were obtained from horses and donkeys on days 0, 7, 14, 30, 90, and 120 following vaccination.

In the VNT, serum was diluted in two steps and mixed with 100–1000 TCID_50_ of EHV-4 before being added to cells that could be infected. The absence of any cell damage showed that neutralization occurred. The lack of cytopathic effect (CPE) signified neutralization. Titers were documented as the maximum serum dilution that entirely obstructed viral multiplication.

### 2.6. Statistical Analysis

All statistical analyses were conducted utilizing Microsoft Excel 365. Viral levels are measured using the Reed–Muench method and reported as log_10_ TCID_50_/cm^3^, with standard deviation (SD) showing how much the values varied. Antibody responses are expressed as mean ± SD in log_2_ titer values. Differences in antibody levels between groups and over time were checked using repeated-measures ANOVA, followed by Tukey’s test to compare multiple groups. Group comparisons (e.g., vaccinated versus control) were assessed using one-way ANOVA when appropriate. A *p*-value less than 0.05 was deemed statistically significant. Confidence intervals (95%) were computed where necessary. All experiments were performed in biological triplicates unless specified differently.

## 3. Results

### 3.1. Preparation and Validation of Experimental Samples for the EHV-4 Vaccine (Lyophilized Culture)

To make the experimental samples of the equine herpesvirus type 4 (EHV-4) vaccine in a dried culture form, a frozen stock of the LT-KK49 testicular cell line (from lamb testis) was thawed and grown in new cultures. The cells were collected by transferring them twice in Eagle’s nutritional medium with 10% fetal bovine serum (FBS), and then were grown in 1.5 L roller bottles.

After a healthy layer of cells formed, the modified harmless strain of EHV-4 was added to the LT-KK49 cell culture and passed through two rounds. Real-time PCR (qPCR) was used to check for the presence of horse herpesvirus type 4 in the virus-filled culture liquids. Cycle threshold (Ct) values were analyzed according to the manufacturer’s guidelines. Samples yielding Ct values within the permissible detection range were verified as EHV-4-positive. Suitable positive and negative controls were incorporated in the experiment to ensure accuracy and eliminate contamination.

Upon confirmation, the virus was amplified in a third passage, resulting in a final culture volume of 2.5 L. The collected viral suspension was frozen and then thawed once to help release the virus, and it was later tested for cleanliness by growing it on special media. No bacterial, fungal, or foreign virus contamination was identified. The viral activity was measured by diluting the samples in single-layer cell cultures kept in penicillin flasks. The infectious virus titer was determined to be 10^6.67^ TCID_50/cm_^3^_._

### 3.2. Biological Activity

The effectiveness of the EHV-4 dry culture vaccine was tested by checking the amount of virus in three types of cells—SPEV (swine embryo kidney), MDBK (Madin–Darby bovine kidney), and LT-KK49 (lamb testis-derived)—and in developing chicken eggs. Samples with viruses were diluted ten times in a row, and the amounts were measured using the Reed–Muench method. Samples containing viruses underwent tenfold serial dilutions, and titers were determined using the Reed–Muench method. The cytopathic effect (CPE) was utilized as the endpoint in cell cultures and observed for 72 h following inoculation. In ECEs, embryonic mortality and lesion formation were assessed over a period of up to 7 days.

Table 1 shows the results of virus titration for each model, highlighting whether cytopathic effects were seen at different dilutions and the calculated viral titers. These data facilitated a comparative examination of substrate sensitivity and viral yield among the evaluated systems.

### 3.3. Vaccine Production, Stabilization, and Lyophilization

#### 3.3.1. Preparation of Viral Biomass

The viral biomass used to make the vaccine was taken from LT-KK49 cell cultures that showed clear signs of damage. Virus-laden culture fluid was harvested once ≥80% of the cell monolayer exhibited discernible cytopathic effects, generally between 72 and 96 h post-infection. The suspension was cleared using low-speed centrifugation and preserved at −80 °C until subsequent processing.

#### 3.3.2. Vaccine Formulation and Lyophilization

The purified viral suspension was utilized to create a liquid vaccine formulation. Aliquots of 1.0 cm^3^ were transferred into sterile glass vials and subsequently freeze-dried using a specialized laboratory-scale lyophilizer. The lyophilization process comprised an initial freezing phase at −40 °C, succeeded by vacuum drying at −50 °C for a duration of 24–36 h. 

Various stabilizing compounds were assessed to assure viral stability throughout drying and storage. Each formulation comprised one or more of the subsequent components: sucrose; peptone; gelatin; lactalbumin hydrolysate; lactose.

#### 3.3.3. Stabilizer Optimization Results

Researchers evaluated various combinations of protective agents to find the ideal stabilizer composition for maintaining viral infectivity during freeze-drying. Each formulation comprised various combinations of sugars and proteins, which are often employed to ensure the stability of viral vaccines. After freeze-drying, the rehydrated vaccine samples were tested in LT-KK49 cell cultures using the TCID_50_ assay, and the amounts of virus were measured using the Reed–Muench method. The viral titers post-lyophilization served as a comparative assessment of stabilizer effectiveness.

Table 2 summarizes the stabilizer compositions, component counts, and associated virus titers. The highest level of viral activity was found in mixtures that included sucrose, gelatin, and lactalbumin hydrolysate.

### 3.4. Stability Evaluation of the Lyophilized EHV-4 Vaccine

#### Accelerated and Real-Time Stability Testing

To see how stable the lyophilized EHV-4 vaccine is when it warms up, vials with the best combination of stabilizers (sucrose, gelatin, and lactalbumin hydrolysate) were stored at three different temperatures: 4 ± 2 °C (in the fridge), 25 ± 2 °C (at room temperature), and 37 ± 2 °C (hotter). Samples were taken out after 1, 3, and 6 months of storage and mixed with a solution to test how strong the virus was, using the TCID_50_ test on LT-KK49 cells. The objective was to assess the degree of potency preservation in conditions mimicking standard storage and possible thermal stress.

The findings, displayed in Table 3, demonstrate a progressive reduction in the virus titer over time at increased temperatures, with negligible loss under refrigeration. The formulation stayed effective at over 5.8 log_10_ TCID_50_/cm^3^ at 25 °C after six months, showing that it is very stable at higher temperatures.

At 4 °C, the vaccine preserved almost 99% of its initial titer for 6 months, demonstrating exceptional cold-chain stability. At 25 °C, a progressive decrease of around 0.35 log_10_ TCID_50_/cm^3^ was noted by month 6 (still within tolerable potency thresholds). At 37 °C, a significant fall in titer (~1.35 log_10_) was observed, indicating that the product should not be subjected to extended heat exposure.

The EHV-4 dry culture vaccine exhibits significant stability at both refrigerated and ambient temperatures for a minimum duration of 6 months. Even though keeping it at 37 °C caused a lot of damage, the formulation is strong enough to handle short periods of being above the best storage temperature, which is important for use in hot or rural areas.

### 3.5. Evaluation of Sterility, Safety, Biological Activity, and Immunogenicity of the EHV-4 Dry Culture Vaccine

#### 3.5.1. Sterility Testing

The sterility of the lyophilized EHV-4 vaccination samples was evaluated using nutritive media, as specified by GOST 28085-89, in conjunction with thioglycollate medium as a comparative fast assay. Vaccine samples were reconstituted in sterile saline and inoculated into meat peptone broth (MPB), meat peptone agar (MPA), meat peptone glucose broth (MPGB), and Sabouraud agar.

Control samples comprised a sterile autoclaved saline solution and a deliberately contaminated vaccine sample injected with secondary microbial flora. Both approaches showed identical sensitivity in identifying microbial and fungal contamination. The vaccine samples and saline controls exhibited no microbial proliferation, hence affirming sterility. The results are summarized in Table 4.

#### 3.5.2. Safety Testing (Revised and Expanded)

The safety of the EHV-4 dry culture vaccine was tested in different types of lab and pet animals to see how well it was accepted and to find any possible side effects. The vaccine was given as an injection into the muscle at 2–3 times the usual dose, depending on the species, to simulate a situation with high exposure. A total of 36 animals were assessed, comprising 15 white mice, 6 rabbits, 6 guinea pigs, 5 donkeys, and 4 horses. Dosages were modified based on body weight and species-specific protocols, varying from 0.5 mL in murine subjects to 10.0 mL in equines.

Post-vaccination, all animals were observed daily for a duration of 14 consecutive days. Observations encompassed body temperature (in rabbits, donkeys, and horses), reactions at the local injection site, appetite, behavior, and survival rates. No animals had systemic or local adverse effects, nor were abnormalities in normal physiological behavior or clinical states seen during the monitoring period.

Table 5 presents the summary data, demonstrating full tolerance and the absence of harmful effects in all examined animal groups.

The results affirm that the EHV-4 dry culture vaccine is safe and well tolerated across many species, with no adverse effects noted, even at increased dosages. The lack of local reaction, systemic symptoms, and death substantiates the vaccine’s non-toxicity and appropriateness for additional efficacy evaluation.

#### 3.5.3. Immunogenicity Testing

The effectiveness of the EHV-4 dry culture vaccine was tested in both small and large animals by checking their virus-specific antibody responses and how well the vaccine worked against challenges. To ensure fair comparisons, the same number of animals was assigned to both the vaccinated and control groups. To facilitate equitable statistical comparisons, identical quantities of animals were allocated to the vaccinated and control groups.

A total of 12 female guinea pigs were enrolled in the study. The vaccinated cohort (*n* = 6) was administered 1000 TCID_50_ of the EHV-4 vaccine via intramuscular injection, whereas the control cohort (*n* = 6) was given a saline placebo. On day 14 post-vaccination, females were mated, and on day 26, all subjects were challenged with 10⁴ TCID_50_ with a severe EHV-4 field strain. Pregnancy outcomes and infant survival were evaluated to determine protective effectiveness.

The results shown in Table 6 indicate that the vaccinated group had better pregnancy retention and significantly higher offspring survival compared to the control group, suggesting that the vaccination offers effective protection.

Vaccinated animals showed complete protection against abortion, while 66.7% of the control group experienced fetal loss post-infection. The survival rate of offspring was also higher in the vaccinated group.

To assess the immunogenicity of the EHV-4 vaccine in target species, 24 animals—12 horses and 12 donkeys—were recruited and equally allocated into vaccinated and control groups. Each vaccinated animal was administered 10 mL of the experimental EHV-4 vaccine via intramuscular injection, while control animals were given an identical volume of saline.

Blood samples were obtained from all animals on days 0, 7, 14, 30, 90, and 120 following vaccination. We measured the amount of virus-fighting antibodies using virus neutralization tests (VNTs) in the flat layers of cells that could be infected by the virus. Neutralizing antibody titers are shown as log_2_; values, indicating the maximum serum dilution that can prevent cytopathic impact. 

Table 7 presents the mean antibody titers over time. Vaccinated equines exhibited a gradual elevation in neutralizing antibodies, reaching a zenith around day 90. No detectable antibodies were identified in any of the control animals.

## 4. Discussion

This paper describes how a freeze-dried vaccine candidate for equine herpesvirus type 4 (EHV-4) was created and tested before clinical trials. This formulation is designed to improve the stability, safety, and effectiveness of EHV-4 vaccination, particularly in areas where keeping vaccines cold is difficult. The results of this study indicate that the experimental vaccine is well tolerated in several species, immunogenic in both small and big animals, and stable during prolonged storage at diverse temperatures.

The EHV-4 virus was propagated utilizing the LT-KK49 cell line, which is generated from lamb testicular tissue. Of the four cell lines tested—LT-KK49, SPEV, MDBK, and developing chicken embryo cells—LT-KK49 showed the best ability to grow the virus and produced the highest amounts, while also causing normal cell changes without much harm to the surrounding cells. These attributes, together with its capacity to scale in roller bottle cultures, rendered it an appropriate selection for generating the viral biomass necessary for vaccine formulation. Although other lines, like MDBK, are frequently utilized in herpesvirus vaccine production, LT-KK49 was chosen solely based on its comparative efficacy under the specific experimental settings. Future comparison research may be necessary for optimizing industrial-scale processes.

The virus was confirmed using real-time PCR, which verified the genetic identity of the EHV-4 strain used to make the vaccine. This step was important for telling EHV-4 apart from the similar but more harmful EHV-1, and for making sure the virus remained unchanged during the production stages.

The vaccine was made stable using a combination of sucrose, gelatin, and lactalbumin hydrolysate, which showed the best ability to maintain strength after freeze-drying compared to the other stabilizers tested. Viral infectivity persisted over 6.0 log_10_ TCID_50_/cm^3^ following six months of storage at 4 °C, with acceptable levels sustained even at 25 °C. Partial stability was maintained at 37 °C, indicating resilience to thermal exposure, a crucial attribute for vaccines deployed in the field. These results corroborate earlier research indicating that sugar–protein matrices successfully preserve viral integrity during freeze-drying and storage.

Safety tests verified the lack of adverse effects in mice, guinea pigs, rabbits, donkeys, and horses, even when the vaccine was provided at doses surpassing the typical immunizing quantity. No indications of local reactivity, alterations in behavior, or mortality were noted throughout the study period. The safety profile is equivalent to or superior to that of current EHV vaccinations in use.

The vaccine showed that it could trigger a strong immune response by producing neutralizing antibodies in horses and donkeys, peaking around day 90 and still being detectable until day 120. Antibody levels were similar to those from a commercial vaccine, suggesting equal or possibly better long-term protection. The levels of antibodies were similar to those produced by a commercial vaccine, suggesting that this vaccine may provide the same or even better long-term protection for the immune system. The guinea pig model further confirmed that the vaccine effectively protects against reproductive issues; vaccinated females had 100% pregnancy success and higher survival rates for their offspring after being exposed to the virus compared to those that were not vaccinated.

Today’s methods for stopping equine herpesvirus infections mainly use inactivated or modified live virus (MLV) vaccines [28], which are mostly offered as single-strain EHV-1 or two-strain EHV-1/4 options [29,30]. These encompass commonly utilized products, such as Pneumabort-K, Prodigy, and Rhinomune. While they do a good job of reducing the seriousness of respiratory diseases and abortions related to EHV-1, they are not very effective at preventing EHV-4. Many research studies have shown that these vaccinations reduce symptoms but do not stop infection or the virus from spreading, especially with EHV-4. Moreover, they typically confer transient immunity, necessitating regular supplements to sustain protective antibody levels; hence, they pose logistical and financial issues for equine management programs [31].

Significantly, there are no authorized commercial vaccines particularly designed for EHV-4, despite its acknowledged position as the principal cause of upper respiratory tract infections in foals and young horses. This indicates a distinct deficiency in current preventive measures, especially in areas or breeding settings where EHV-1-related consequences like miscarriage or neurological disorders are not the foremost issue.

Alongside licensed pharmaceuticals, other experimental vaccine platforms have been explored. This collection includes genetically modified deletion variants of EHV-1, including the ORF2-deficient Ab4ΔORF2 strain, which has demonstrated encouraging outcomes in diminishing viremia and viral shedding in equines [32]. Additional research has investigated the capacity of DNA vaccines and recombinant viral vectors to elicit more extensive immune responses. A recent systematic review determined that both commercial and experimental EHV vaccinations typically provide partial protection and exhibit low efficiency in preventing transmission or clinical illness, particularly in field situations [24,31,33].

Unlike traditional methods, the vaccine described here comes from a harmless EHV-4 strain, made specifically to prevent EHV-4 and preserved through freeze-drying. This emphasis on EHV-4 alone presents prospective benefits for tailored immune priming and practical implementation. The freeze-dried version stays stable at room temperature, allowing for longer storage and making it easier to use in remote areas that do not have good refrigeration.

The good immune response and safety shown in horses, donkeys, and lab animals in this study suggest that this candidate could be a useful alternative or addition to current bivalent vaccines, especially in areas where EHV-4 is the main strain. Continuing to evaluate it in real-world settings and undertaking more comparison studies will be important to understand its role in overall EHV vaccination efforts. 

## 5. Conclusions

A dry culture vaccine for horse herpesvirus type 4 (EHV-4) was formulated and assessed in laboratory settings. The vaccine was made using LT-KK49 cell culture, mixed with a special formula of sucrose, gelatin, and lactalbumin, and then dried out. It exhibited stability across diverse storage settings and did not elicit adverse reactions in any of the examined animal species. Immunogenicity was validated in guinea pigs, horses, and donkeys, whereas protective effectiveness was established in a guinea pig challenge paradigm. The findings offer information for additional assessment and possible implementation in EHV-4-preventive initiatives.

## Figures and Tables

**Table 1:** Virus titer of the EHV-4 vaccine in various biological models.

Cell Model/Substrate	10^−^⁴	10^−^⁵	10^−^⁶	10^−^⁷	TCID_50_ (log_10_ TCID_50_/cm^3^ ± SD)
SPEV	++++	++++	+−−−	−−−−	5.25 ± 0.18
MDBK	++++	++++	++−−	−−−−	5.75 ± 0.12
LT-KK49	++++	++++	++++	−−−−	6.25 ± 0.09
DCE	++++	++++	−−−−	−−−−	Not determined

Note: “+” indicates presence of CPE or embryonic death; “−” indicates absence. Titers calculated using Reed–Muench; SD = standard deviation.

**Table 2:** Effect of stabilizer composition on post-lyophilization viral titer.

Stabilizer Composition	Composition (% *w*/*v*)	Post-Lyophilization Virus Titer (log_10_ TCID_50_/cm^3^ ± SD)
Sucrose + Lactalbumin hydrolysate	Sucrose (5%) + Lactalbumin hydrolysate (2.5%) + WFI up to 100%	6.15 ± 0.10
Peptone + Gelatin	Peptone (3%) + Gelatin (2%) + WFI up to 100%	5.80 ± 0.12
Lactose + Lactalbumin hydrolysate	Lactose (4%) + Lactalbumin hydrolysate (2%) + WFI up to 100%	5.92 ± 0.15
Sucrose + Gelatin + Lactalbumin hydrolysate	Sucrose (4%) + Gelatin (1.5%) + Lactalbumin hydrolysate (2%) + WFI up to 100%	6.20 ± 0.08
Peptone + Lactose	Peptone (3%) + Lactose (4%) + WFI up to 100%	5.65 ± 0.14
No stabilizer (control)	WFI only (100%)	4.90 ± 0.18

Note: WFI—water for injection.

**Table 3:** Residual viral titers (log_10_ TCID_50_/cm^3^) under different storage conditions over time.

Storage Temp	Month 1	Month 3	Month 6
4 °C	6.15 ± 0.08	6.10 ± 0.07	6.05 ± 0.10
25 °C	6.05 ± 0.09	5.95 ± 0.10	5.80 ± 0.12
37 °C	5.80 ± 0.11	5.40 ± 0.15	4.85 ± 0.18

**Table 4:** Results of vaccine sterility testing on various media.

Sample Type	MPB	MPA	MPGB	Sabouraud	Thioglycollate	Conclusion
Vaccine (mean sample)	0/4	0/4	0/2	0/4	0/4	Sterile
Autoclaved saline	0/4	0/4	0/2	0/4	0/4	Sterile
Contaminated control	2/4 (day 3–5)	1/4 (passage 2)	0/2	1/4 (day 15)	4/4 (day 2–3)	Contaminated with bacteria and fungi

Note: Numerator indicates positive contamination samples; denominator indicates total seeded samples.

**Table 5:** Clinical observation results following safety testing of the EHV-4 vaccine.

Species	No. of Animals	Dose per Animal (mL)	Local Reaction	Behavioral Changes	Temperature Range (°C)	Mortality	Overall Status
White mice	15	0.5	None	None	Not monitored	0/15	Normal
Guinea pigs	6	1.0	None	None	Not monitored	0/6	Normal
Rabbits	6	5.0	None	None	38.7–39.6	0/6	Normal
Donkeys	5	10.0	None	None	37.0–37.8	0/5	Normal
Horses (mares)	4	10.0	None	None	37.2–38.4	0/4	Normal

**Table 6:** Pregnancy and fetal outcomes in guinea pigs after challenge.

Group	Pregnant (*n*)	Abortions (*n*)	Healthy Offspring (*n*)	Surviving Offspring (*n*)
Vaccinated	6	0	15	14
Unvaccinated	6	4	5	3

**Table 7:** Mean antibody titers (log_2_) in horses and donkeys following immunization.

Group	*n*	Day 0	Day 7	Day 14	Day 30	Day 90	Day 120
Horses—Vaccinated	6	0	0.6 ± 0.05	1.8 ± 0.04	4.0 ± 0.08	5.0 ± 0.15	3.6 ± 0.19
Horses—Control	6	0	n.d.	n.d.	0	n.d.	0
Donkeys—Vaccinated	6	0	0.4 ± 0.06	1.3 ± 0.07	3.3 ± 0.11	3.9 ± 0.13	2.6 ± 0.16
Donkeys—Control	6	0	n.d.	n.d.	0	n.d.	0

Note: “n.d.” = not detected (antibodies below assay threshold).

## Data Availability

The original contributions presented in this study are included in the article. Further inquiries can be directed to the corresponding author(s).
